# Considerations related to the use of short neuropeptide promoters in viral vectors targeting hypothalamic neurons

**DOI:** 10.1038/s41598-019-47417-9

**Published:** 2019-07-31

**Authors:** N. Kakava-Georgiadou, C. Bullich-Vilarrubias, M. M. Zwartkruis, M. C. M Luijendijk, K. M. Garner, R. A. H. Adan

**Affiliations:** 1 Department of Translational Neuroscience, Division of Neuroscience, UMC Utrecht Brain Center, University Medical Center Utrecht, Utrecht, The Netherlands; 2 Master’s Program Neuroscience and Cognition, Utrecht University, Utrecht, The Netherlands; 3 Institute of Neuroscience and Physiology, The Sahlgrenska Academy at the University of Gothenburg, Gothenburg, Sweden

**Keywords:** Neural circuits, Genetic vectors

## Abstract

Targeting specific neuronal cell types is a major challenge for unraveling their function and utilizing specific cells for gene therapy strategies. Viral vector tools are widely used to target specific cells or circuits for these purposes. Here, we use viral vectors with short promoters of neuropeptide genes to target distinct neuronal populations in the hypothalamus of rats and mice. We show that lowering the amount of genomic copies is effective in increasing specificity of a melanin-concentrating hormone promoter. However, since too low titers reduce transduction efficacy, there is an optimal titer for achieving high specificity and sufficient efficacy. Other previously identified neuropeptide promoters as those for oxytocin and orexin require further sequence optimization to increase target specificity. We conclude that promoter-driven viral vectors should be used with caution in order to target cells specifically.

## Introduction

The central nervous system is characterized by enormous diversity in cell types and projections. Traditionally, cell types are defined by distinct expression of marker genes. Within a brain region, distinct cell types play different roles in regulating functions and behaviors. For example, in the arcuate nucleus of the hypothalamus Agrp/Npy neurons are molecularly distinct from POMC/CART and they have opposing effects on food intake and energy expenditure^1,2^. In the lateral hypothalamus, orexin and melanin-concentrating hormone (MCH) neurons both promote the consumption of palatable food, but affect different aspects of food intake: orexin neurons by promoting food seeking and motivation for food reward and MCH by prolonging consumption of palatable food^3^. Loss of limited numbers of specific neurons has the potential to result in disease. For instance, loss of orexin neurons underlies narcolepsy^4^ and Parkinson’s disease is characterized by loss of dopamine neurons^5^.

Targeting distinct cell types in specific areas of the brain is critical for the understanding of their specific function and role in behavior as well as for utilizing specific cells for therapeutic interventions in humans.

In preclinical studies, viral vectors are also used for manipulation and recording of neuronal activity, neuronal silencing and overexpression or knock-down of desired proteins in specific cell types or circuits. A straightforward approach to target specific cells with these tools is to generate mice or rats in which recombinases, such as Cre and Flp^6^, are expressed in a locus of a marker gene that is selectively expressed in those cells. Viral vectors carrying transgenes, of which the expression depends on these recombinases, can be injected in brain regions and target the cells of interest. Nevertheless, when a combination of tools needs to be used, for example to simultaneously activate a cell type and record from another one, generation and breeding of multi-transgenic animals becomes a hurdle. Moreover, the use of wild type instead of transgenic animals makes these tools directly applicable to higher primates. A way to limit the use of transgenic animals is to develop viral vectors that use a short promoter of a marker gene to target a specific cell type.

A few neuropeptide promoters have been used in the literature to record from neurons *in vitro* or activate them with optogenetics and chemogenetics. The most widely used promoter to target a neuronal subtype is CamKIIα, which targets expression in (subsets of) glutamatergic cells^7^.

Regarding the hypothalamus, melanin-concentrating hormone (MCH) neurons have been extensively targeted with adeno-associated viruses (AAVs) that express genetic tools under the control of the MCH promoter (MCHpr). van den Pol *et al*.^8^ recorded neuronal activity of MCH neurons tagged with GFP under the control of the rat MCHpr. In Konadhode *et al*.^9^, MCH neurons were targeted with the light-sensitive channel Channel Rhodopsin 2 (ChR2) which allowed their transient activation for milliseconds and their role in sleep was investigated. In Noble *et al*.^10^, the role of MCH neurons in feeding was investigated by expressing a designer receptor exclusively activated by designer drugs (DREADD) under the control of the rat MCHpr, which when activated by Clozapine N-Oxide (CNO) transiently increases neuronal firing for hours. Moreover, different fragments of the mouse oxytocin promoter (OXTpr) have been used to target oxytocin-expressing cells in the PVN with ChR2 and DREADD and study their role in feeding and autism respectively^11,12^. Finally, the human Orexin promoter has been used to record from Orexin neurons in the hypothalamus^13^.

Despite the availability of promoters short enough to be accommodated in AAV transgene cassettes and specific enough to restrict expression in cell-types, they have not been very widely used so far. A cause of this might be the low efficiency of viral transduction, that can be improved by using different serotypes or serotype-“hybrids” of AAV and increased titers. On top of that, promoter-driven constructs can result in off-target expression when a lot of copies are present in a cell^14^. We aimed to investigate this further by assessing outcomes of viral transduction, such as specificity and efficiency of expression and question the implications that these outcomes could have on functionality by testing promoters that have been used to target cells in the hypothalamus: MCH neurons in the LH, Orexin neurons in the LH and perifornical area and Oxytocin neurons in the paraventricular nucleus (PVN) of the hypothalamus.

## Results

### Established neuropeptide promoters lead to non-specific expression at high titers

In order to achieve viral transfer in the hypothalamus we determined the ability of already established cell-specific promoters to drive specific expression of optogenetic and chemogenetic tools at titers (amount of genomic copies) commonly used in the literature.

**Figure 1:**
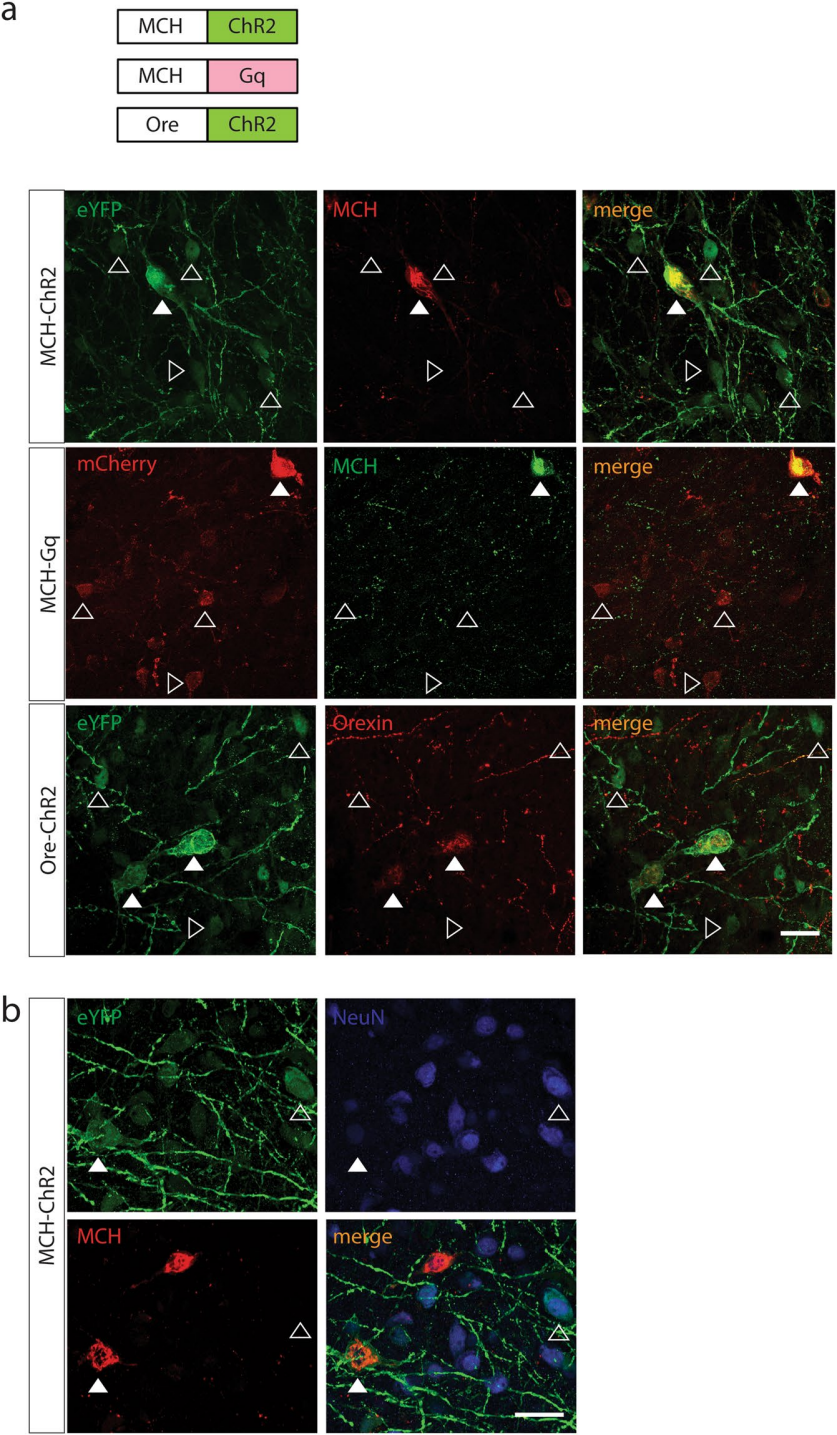
Established neuropeptide promoters driving expression of transgenes in the hypothalamus. (**A**) Top: The rat MCH promoter^8^ driving expression of ChR2:eYFP (MCH-ChR2:eYFP): co-staining for MCH (red) and eYFP (green); Middle: The rat MCH promoter^8^ driving expression of Gq:mCherry(MCH-Gq:mCherry): co-staining for MCH (green) and mCherry (red),eYFP or mCherry expression in MCH^+^ cells (full white arrows), expression in MCH^−^ cells (empty white arrows); Bottom: The human Orexin promoter^13^ driving expression of ChR2:EYFP (Ore-ChR2:eYFP) in the mouse hypothalamus, co-staining for Orexin (red) and eYFP (green); eYFP expression in Orexin^+^ cells (full white arrows), expression in Orexin^−^ cells (empty white arrows). (**B**) MCH-ChR2:eYFP injected in the rat hypothalamus; co-staining for MCH (red), NeuN (blue), eYFP (green); eYFP^+^NeuN^+^ in MCH^+^ cells (full white arrow), eYFP^+^NeuN^+^ in MCH^−^ cells (empty white arrow). Scale bars: 20 µm.

To this end, we utilized an AAV carrying the rat 463 bp MCH promoter (rMCHpr), first established in van den Pol *et al*.^8^, driving expression of Channel Rhodopsin 2 fused with eYFP (**MCH-ChR2:eYFP**) as previously used in Konadhode *et al*.^9^, injected in the rat lateral hypothalamus at 3 × 10^9^ g.c. per µL. Moreover, we created an AAV with the rMCHpr driving expression of the DREADD receptor hM3D(Gq) fused with mCherry (**MCH-Gq:mCherry**) and injected it in the rat lateral hypothalamus at 0.3 × 10^9^ g.c. per µL. Both of these constructs resulted in high expression of fluorophores in MCH^+^ cells; however, there was also expression in many MCH^−^ cells (top and middle panel, Fig. 1a). Non-specific expression of fluorophores was clearly observed in areas poor in MCH^+^ cells (Fig. S1a), as well as in sections caudal to the injection site, in which there are almost no MCH cells (Fig. S1b).

To assess whether the non-specific expression observed is due to the AAV capsid of serotype 5, we also injected the MCH-ChR2:eYFP construct packaged in capsids of serotypes AAV1 and AAV2 in pilot experiments. With AAV1 at the same titer (3 × 10^9^ g.c. per µL) we observed similar levels of non-specific expression (data not shown), whereas AAV2 was produced at a low titer (0.3 × 10^9^ g.c. per µL) and transduced very few cells in the hypothalamus. Therefore, we assumed that there is no difference regarding specificity between AAV1 and AAV5 and decided to package all the AAV constructs in this study in AAV capsid of serotype 5.

When injecting MCH-ChR2:eYFP unilaterally into the ventral tegmental area (VTA), where no MCH neurons are present, eYFP^+^ cells were observed (Fig. S2a).

We also cloned a human 1.3 kb-long Orexin promoter (Orepr), as used in Saito *et al*.^13^, in front of ChR2:eYFP (**Ore-ChR2:eYFP**), packaged it in AAV and injected it in the mouse hypothalamus at 1 × 10^9^ g.c. per µL. Expression of eYFP was not only observed in Orexin^+^ cells but also in Orexin^−^ cells (bottom panel, Fig. 1a).

We next questioned the identity of the cells where non-specific expression was observed. We did not observe co-localization of eYFP with Orexin on tissue of brains injected with MCH-ChR2:EYFP (Fig. S2b). Therefore, we evaluated whether these cells were neurons. Qualitative assessment revealed that there are MCH^−^EYFP^+^ cells that also express NeuN (Fig. 1b).

Considering these results, we reasoned that weak promoter activity of promoters in non-specific cells resulted in non-specific expression of transgenes, when a high number of genomic copies ends up in a cell. We therefore determined whether lowering the titer of the injected AAV vectors increases specificity.

### Titer affects specificity and efficiency of promoter-driven expression

We decreased the titer of MCH-ChR2:eYFP from 3.0 to 0.3, 0.1 and 0.03 × 10^9^ g.c. per µL and quantified the specificity and efficiency of these titers to target MCH neurons in the lateral hypothalamus. We hypothesized that by lowering the amount of genomic copies that enters a cell, specificity would be increased, whereas efficiency would be decreased.

**Figure 2:**
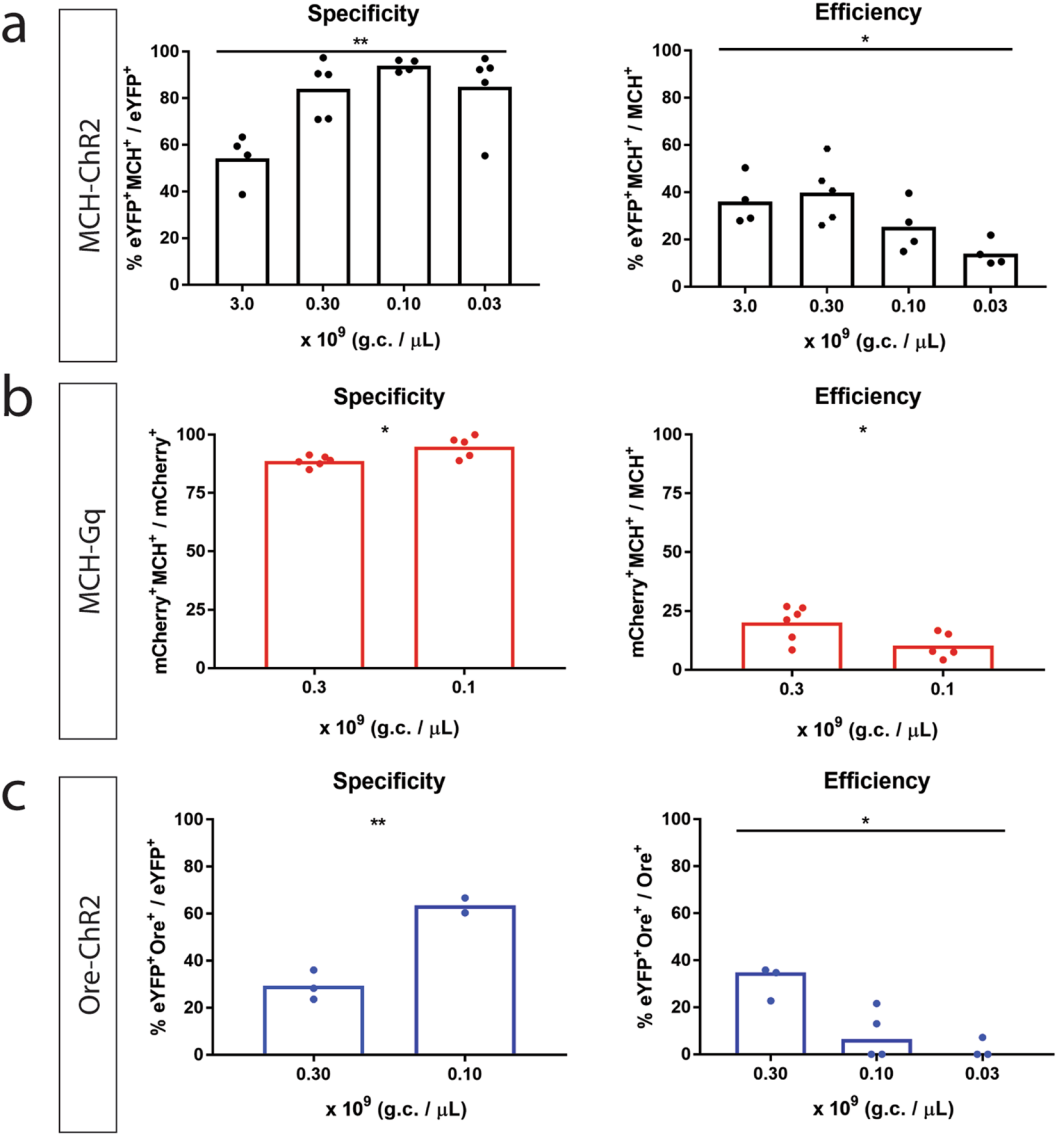
Effect of titer on specificity and efficiency of promoter-driven viral constructs. (**A**) MCH-ChR2:eYFP injected at 3.0 to 0.3, 0.1 and 0.03 × 10^9 ^ g.c. per µL in the lateral hypothalamus of rats; Left: Specificity as % eYFP^+^MCH^+^/eYFP^+^ cells in the lateral hypothalamus (F (3, 14) = 7,997,n = 4–5 per group, *p* = 0.0024), Right: Efficiency as % eYFP^+^MCH^+^/MCH^+^ cells in the lateral hypothalamus (one-way ANOVA: F(3,13) = 5,253,n = 4–5 per group, *p* = 0.0136); (**B**) MCH-Gq:mCherry at 0.3 and 0.1 × 10^9^ g.c. per µL injected in the lateral hypothalamus of rats; Left: Specificity as % mCherry^+^MCH^+^/mCherry^+^ cells in the lateral hypothalamus (unpaired *t*-test, t(9) = 2,9, n = 5–6 per group, *p* = 0.0176),Right: Efficiency as % mCherry^+^MCH^+^/MCH^+^ cells in the lateral hypothalamus (unpaired *t*-test, t(9) = 2,455,n = 5–6 per group, *p* = 0.0364); (**C)** Ore-ChR2:eYFP injected at 0.3, 0.1 and 0.03 × 10^9^ g.c. per µL in the hypothalamus of mice; Left: Specificity as % eYFP^+^Orexin^+^/eYFP^+^ cells in the hypothalamus (unpaired *t*-test, t(3) = 6,565, *p* = 0.0072), at 0.03 × 10^9^ g.c. per µL 2 out of 3 mice didn’t show expression, thus specificity is not included in the graph, Right: Efficiency as % eYFP^+^Orexin^+^/Orexin^+^ cells in the hypothalamus (Kruskal-Wallis test: χ^2^(3, N = 10) = 6,368, *p* = 0.0219); All graphs represent mean, except for right panel of C, which represents median; *P < 0.05, **P < 0.01; Dots represent individual animals.

Indeed, lowering the titer of MCH-ChR2:eYFP significantly increased specificity (one-way ANOVA: F (3, 14) = 7,997, *p* = 0.0024, n = 4–5 per group, Fig. 2a, left), and decreased efficiency (one-way ANOVA: F(3,13) = 5,253, *p* = 0.0136, n = 4–5 per group, Fig. 2a, right).Moreover, at low titers, there was no expression of eYFP in caudal sections (Fig. S1c).

MCH-ChR2:eYFP had similar efficiency to target MCH neurons at 0.3 × 10^9^ g.c. per µL compared to 3.0 × 10^9^ g.c. per µL (0.3: 39,91% ± 5,782, 3.0: 36,05% ± 5,165, mean ± SEM). However, specificity of MCH-ChR2:eYFP reached maximum levels at 0.1 × 10^9^ g.c. per µL (93,94% ± 1,266, mean ± SEM), whereas efficiency became low (25,3% ± 5,43, mean ± SEM).

Therefore, we decided to use titers of 0.1 and 0.3 × 10^9^ g.c. per µL in order to explore if there is an effect of titer on specificity and efficiency for MCH-Gq:mCherry and Ore-ChR2:eYFP.

A 3-fold decrease of the titer of MCH-Gq:mCherry from 0.3 to 0.1 × 10^9^ g.c. per µL, significantly increased specificity (unpaired *t*-test, t(9) = 2,9, *p* = 0.0176, n = 5–6 per group, Fig. 2b, left) but decreased efficiency (unpaired *t*-test, t(9) = 2,455, *p* = 0.0364, n = 5–6 per group, Fig. 2b, right).

Furthermore, we targeted the hypothalamus of mice with Ore-ChR2:EYFP at titers of 0.3 and 0.1 × 10^9^ g.c. per µL. Only 2 out of 4 mice injected with the lowest titer showed expression. Based on the results from the mice that showed expression, we found that lowering the titer increased specificity (unpaired *t*-test, t(3) = 6,565, *p* = 0.0072, Fig. 2c, left). However, specificity was low even at levels of 0.1 × 10^9^ g.c. per µL, in contrast with our findings with MCHpr. Therefore, we decided to decrease the titer to 0.03 × 10^9^ g.c. per µL in order to determine if specificity would increase further. However, from 3 mice injected only one showed expression with specificity of 51,11% and efficiency of 12,70%. Overall, across all three titers injected - including the animals that showed no expression - efficiency decreased substantially (Kruskal-Wallis test: χ^2^(3, N = 10) = 6,368, *p* = 0.0219, Fig. 2c, right).

Overall, these results show that lowering the titer of viral vectors with promoter-driven transgenes leads to an increase in specificity, while reducing efficiency to target specific cells.

### Non-specific expression driven by a short promoter is lower than in properly-targeted specific cells

Since we found that reducing titer increases specificity of promoter-driven expression of ChR2:eYFP and Gq:mCherry, we determined whether this affected expression level.

**Figure 3:**
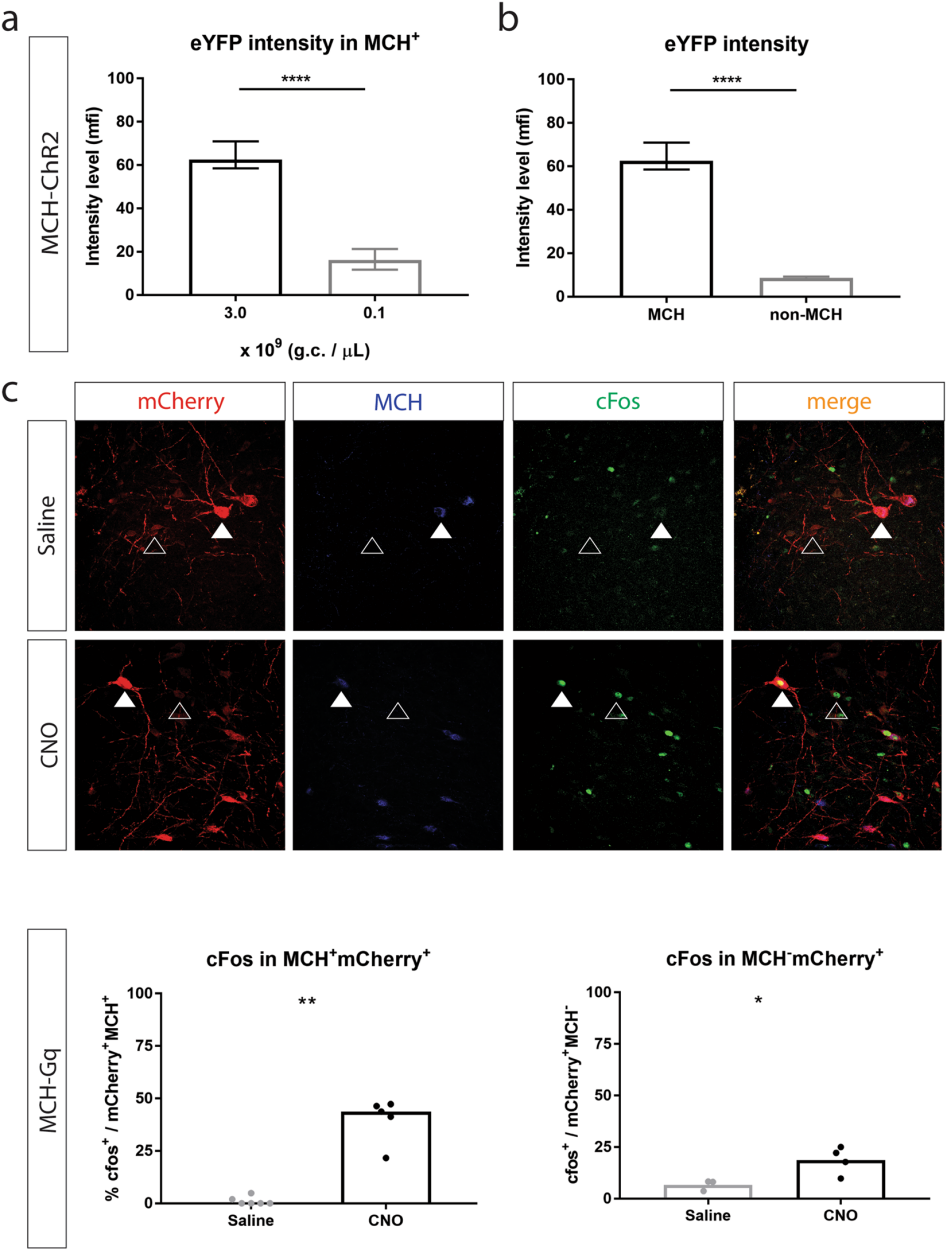
Non-specific expression of transgenes is lower than expression in specific cells. (**A**) Expression levels of eYFP, measured as mean fluorescence intensity (mfi), in MCH^+^ cells at 3.0 and 0.1 × 10^9 ^ g.c. per µL of MCH-ChR2:eYFP (Mann-whitney *u*-test: U = 2924, *p* < 0.0001); (**B**) Expression levels of eYFP, measured as mean fluorescence intensity (mfi), in MCH^+^ and MCH^−^ cells at 3.0 × 10^9^ g.c. per µL of MCH-ChR2:eYFP (Mann-whitney *u*-test: U = 120, *p* < 0.0001); (**C**) cFos expression in mCherry^+ ^cells after CNO and Saline administration in rats injected with MCH-Gq:mCherry at 0.1–3 × 10^9^ g.c. per µL in the lateral hypothalamus; Top: Co-staining for Gq:mCherry (red), MCH (blue), cFos (green) after Saline administration, cFos^−^Gq^+^MCH^+^ (full white arrows), cFos^−^Gq^+^MCH^−^ (empty white arrows); Middle: Co-staining for Gq:mCherry (red), MCH (blue), cFos (green) after CNO administration, cFos^+^Gq^+^MCH^+^ (full white arrows), cFos^+^Gq^+^MCH^−^ (empty white arrows); Bottom, left: % cFos^+^ cells within mCherry^+^MCH^+^ cells after Saline and CNO administration (Mann-whitney *u*-test: U = 0, n = 5–6 per group, *p* = 0.0043); Bottom, right: % cFos^+^ cells within mCherry^+^MCH^−^ cells after Saline and CNO administration (unpaired *t*-test: t(5) = 2,89, n = 3–4 per group, *p* = 0.0342); Scale bar: 50 µm; Graphs represent: A) median ± 95% CI, B) median ± 95% CI, C) Left: median, Right: mean; *P < 0.05, **P < 0.01, ****P < 0.0001; Dots represent individual animals.

We observed that when lowering the titer from 3.0 to 0.1 × 10^9^ g.c. per µL, at which specificity becomes very high (94,61% ± 2,99, mean ± SEM), the intensity of expression of eYFP in MCH^+^ cells decreased. In particular, at 3.0 × 10^9 ^g.c. per µL, mean fluorescence intensity of eYFP in MCH^+^ cells is significantly lower compared to the intensity of eYFP in MCH^+^ cells when injecting at 0.1 × 10^9^ g.c. per µL (Mann-whitney *u*-test: U = 2924, *p* < 0.0001, Fig. 3a).

When using MCH-ChR2:eYFP at a high titer (3.0 × 10^9^ g.c. per µL), specificity of targeting MCH^+^ cells was 59,52% ± 3,88 (mean ± SEM). However, we noticed that the level of eYFP expression in MCH^−^ cells was much lower than in MCH^+^ cells. More specifically, mean fluorescence intensity (mfi) of eYFP in MCH^+^ cells was significantly higher than in MCH^−^ cells (Mann-whitney *u*-test: U = 120, *p* < 0.0001, Fig. 3b). This was also the case when injecting other constructs at high titers (see Fig. 1a,b).

Even though levels of off-target transgene expression are very low, manipulation of neuronal activity or signal coming from these cells might have implications for interpretation of results when promoter-driven transgenes are used *in vivo* or *in vitro*. Therefore, we aimed to investigate whether there is activation of MCH^−^ cells, as a result of non-specific expression of transgenes.

Animals that were injected with MCH-Gq:mCherry (Fig. 2b), received CNO or saline 2 hours prior to sacrifice. We examined expression of immediately early gene cFos in MCH^+^mCherry^+^ and MCH^−^mCherry^+^ cells after CNO and Saline administration (Fig. 3c, top & middle). After CNO administration the percentage of cFos^+^ cells within MCH^+^mCherry^+^ cells was significantly higher compared to Saline administration (Mann-whitney *u*-test: U = 0, *p* = 0.0043, n = 5–6 per group, Fig. 3c, bottom left). Additionally, after CNO administration the percentage of cFos^+^ cells within MCH^−^Gq^+^ cells was significantly higher compared to Saline administration (unpaired *t*-test: t(5) = 2,89, n = 3–4 per group, *p* = 0.0342, Fig. 3c, bottom right). Four animals were excluded in the latter analysis, because the amount of MCH^−^mCherry^+^ cells was not sufficient enough to include in quantifications (since specificity was very high).

### Promoter-driven Cre leads to non-specific expression at all titers

Expression of Cre recombinase in combination with Cre-dependent (floxed) constructs or conditional mouse lines would potentially more efficiently transduce neurons because, in theory, one molecule of Cre is sufficient to recombine many copies of the Cre-dependent transgenes. We reasoned that if very specific expression of Cre was achieved at low level, this would be sufficient to recombine transgenes under the control of strong promoters so that high levels of expression would be reached.

**Figure 4:**
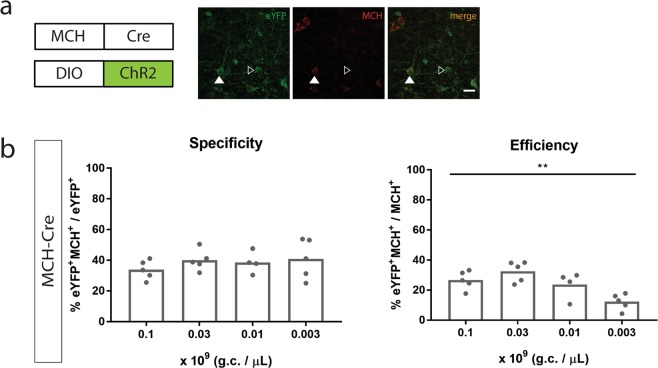
MCH promoter-driven Cre with Cre-dependent (DIO-)ChR2:eYFP. (**A**) The rat MCH promoter driving expression of Cre injected with DIO-ChR2:eYFP in the rat lateral hypothalamus; co-staining for MCH (red) and eYFP (green), eYFP expression in MCH^+^ cells (full white arrows), expression in MCH^−^ cells (empty white arrows); (**B**) MCH-Cre injected in the lateral hypothalamus at 0.1, 0.03, 0.01 and 0.003 × 10^9 ^g.c. per µL with DIO-ChR2:eYFP at 1.0 × 10^9 ^ g.c. per µL; Left: Specificity as % eYFP^+^MCH^+^/eYFP^+^ (one-way ANOVA, F (3, 15) = 0,6473, n = 4–5 per group, *p* = 0.5967); Right: Efficiency as % eYFP^+^MCH^+^/MCH^+^ (one way ANOVA: F (3, 15) = 7,87, n = 4–5 per group, *p* = 0.022); Scale bar: 30 µm; All graphs represent mean; **P < 0.01; Dots represent individual animals.

To test this, we combined injections of rMCHpr driving Cre (MCH-Cre) with a Cre-dependent ChR2:eYFP (DIO-ChR2:eYFP) in the lateral hypothalamus of rats at the two lowest titers that we previously injected MCH-ChR2:eYFP with: 0.1 and 0.03 × 10^9^ g.c. per µL as well as two lower titers: 0.01 and 0.003 × 10^9^ g.c. per µL (titer of DIO-ChR2:eYFP was 1 × 10^9^ g.c. per µL). Serial dilutions of titer did not affect specificity (one-way ANOVA, F (3, 15) = 0,6473, n = 4–5 per group, *p* = 0.5967, Fig. 4, left), whereas efficiency decreased (one-way ANOVA: F (3, 15) = 7,87, n = 4–5 per group, *p* = 0.022, Fig. 4, right).

Additionally, we compared the specificity and efficiency of MCH-Cre & DIO-ChR2:eYFP with MCH-ChR2:eYFP, both injected at 0.1 and 0.03 × 10^9^ g.c. per µL. MCH-Cre & DIO-ChR2:eYFP were significantly less specific to target MCH neurons compared to MCH-ChR2:eYFP at both titers (two-way ANOVA; effect of construct: F(1,15) = 129,7, *p* < 0.0001; post-hoc Sidak’s test, *p* < 0.0001 for 0.1, *p* < 0.0001 for 0.03, Figs 2a and 4). Moreover, MCH-Cre & DIO-ChR2:eYFP showed higher efficiency to target MCH neurons than MCH-ChR2:eYFP (two-way ANOVA; effect of construct: F(1,15) = 7,801, *p* = 0.0144; construct x titer interaction: F (1, 14) = 5,766, *p* = 0.0308, Figs 2a and 4) and this decrease was driven by the titer of 0.03 × 10^9 ^ g.c. per µL (post-hoc Sidak’s test, *p* = 0.9541 for 0.1, *p* = 0.0050 for 0.03). The total number of eYFP^+^ cells transduced with MCH-Cre & DIO-ChR2:EYFP was higher compared to the total number transduced with MCH-ChR2 at the common titers injected (0.1 and 0.03 × 10^9^ g.c. per µL) (Fig. S3a).

We also injected a mouse Oxytocin promoter^11^ driving Cre into the PVN of Rosa26-LSL-YFP mice. A titer of 0.8 × 10^9 ^ g.c. per µL resulted in robust non-specific expression and a low titer of 0.05 × 10^9^ g.c. per µL resulted in very low and still non-specific expression (Fig. S3b).

## Discussion

Short neuropeptide promoters remain attractive tools to target expression to specific cells when using viral vectors. To evaluate and further develop these tools, we here determined specificity and efficiency of a selection of well known neuropeptide promoters.

We showed that constructs that are driven by established cell-specific promoters lead to off-target expression of transgenes in cells when injected at high titers. In particular, we targeted MCH cells in the rat lateral hypothalamus with cDNAs used for optogenetics and chemogenetics under the control of the rat MCH promoter^8^, and showed that ChR2:eYFP and Gq:mCherry were expressed in both MCH^+^ and MCH^−^ cells. Similarly, when targeting orexin cells in the mouse hypothalamus with ChR2:eYFP under the control of the human orexin promoter^13^, expression of ChR2:eYFP was also found in Orexin^−^ cells (Fig. 1a). Off-target expression is likely a result of the high titer injected, because weak promoter activity accumulates when too many genomic copies of a transgene end up in a cell. Indeed, in Fig. 2 we show that specificity and efficiency of expression is dependent on the amount of genomic copies injected. When titer is lowered, specificity increases and efficiency decreases.

Even though these promoters have been used in the literature and evidence was provided that they were specific, we observed non-specific expression. In van den Pol *et al*.^8^, 294 out of 300 GFP + cells (98%) also showed MCH immunoreactivity when MCH-GFP in AAV capsid of serotype 2 was injected in the rat lateral hypothalamus, and there was no GFP signal detected neither in regions where there are no MCH cells or next to the needle tract. Similarly, in Konadhode *et al*.^9^, all eYFP^+^ neurons were also positive for MCH (100%) when MCH-ChR2:eYFP in AAV capsid of serotype 5 was injected in the hypothalamus of mice. In Saito *et al*.^13^, the human orexin promoter was used to drive expression of tdTomato in orexin neurons in the mouse hypothalamus in an AAV capsid of serotype 2. Almost all (>97%) tdTomato-expressing neurons were positive for Orexin.

There are several reasons that could explain why there is a discrepancy between our observations and the literature. First, what is common in all three papers cited, is that no immunohistochemical detection method was used to detect GFP, eYFP and tdTomato respectively, and rather quantifications were made with observing the endogenous signal of these fluorescent proteins. Therefore, there is a possibility that low expression levels remained undetected. We used antibodies against the fluorophores expressed under the control of the neuropeptide promoters, in order become more sensitive to detect expression. We indeed found that the eYFP signal coming from MCH^−^ cells has lower intensity than that coming from MCH^+^ cells (Fig. 4b), which we discuss later. We exclude the possibility of background signal resulting in counting a cell as positive, because with lower titers we reached high levels of specificity. Moreover, even though there is highly non-specific expression (around 60% of eYFP^+^ cells are also MCH^+^) in brains injected with MCH-ChR2:eYFP at 3.0 × 10^9 ^ g.c. per µL, we did not find any co-localization of eYFP and Orexin. Perhaps there is high transcriptional repression of the promoter of the preproMCH gene in orexin cells.

It is worth to discuss that these data are not always directly comparable with the results of other labs that have tested AAV constructs with the same neuropeptide promoters, since the methods used for production, purification and titration of the viral particles and genomic copies differ and therefore the amount of genomic copies injected in the brain can be difficult to compare between studies. For example, considering titration methods, van den Pol *et al*.^8^ measured the number of DNase resistant particles by real-time PCR, Konadhode *et al*.^9^ used dot plot hybridization, whereas we used real-time PCR with primers binding on the wPRE element. Thus, calculated and real amounts of genomic copies may differ between labs which could explain the discrepancies in results.

Another reason that could have led to a difference in our findings could be the choice of AAV capsid, since it influences the uptake of AAVs in specific cells. We packaged all AAVs in capsid of serotype 5, whereas van den Pol^8^ and Saito^13^ used serotype 2. AAV with capsid of serotype 2 is known to transduce smaller areas and infect different cells than that of serotype 5^16–18^. Therefore, we might have infected many more and different types of cells compared to previous studies, even though we used similar titers. Unfortunately, when injecting MCH-ChR2:eYFP packaged in AAV capsid of serotype 2 the efficiency was very low and we could hardly detect eYFP^+^ positive cells, which compromised assessment of the effect of serotype on specificity.

We also noticed that, even though with the rat MCH promoter specific expression was obtained with MCH-ChR2:eYFP and MCH-Gq:mCherry at titer of 0.1 × 10^9^ g.c. per µL, this was not the case when Ore-ChR2:eYFP was injected. When the human orexin promoter was used, even though lowering the titer increased specificity, efficiency was decreased so much that expression of eYFP reached undetectable levels in many of the animals. We speculate that because a human Orexin promoter was used in a different species, it might not possess all the necessary regulatory elements to restrict expression in orexin cells in mice. It is also worth to discuss that the 1.3 kb human orexin promoter that was used by Saito *et al*.^13^ and us, does not contain an important regulatory element, defined as “OE2” in the literature^19^. Transgenic mice with endogenous expression of LacZ under the control of the human 3.2 kb orexin promoter, which includes OE2, show restricted expression of LacZ in orexin neurons of the LH, whereas the 1.3 kb promoter used in the same paradigm results in off-target expression of LacZ in the Arcuate nucleus. Therefore, the human orexin promoter that we used might not have the ability to restrict expression exclusively in orexin neurons.

Furthermore, we cannot exclude the possibility that with lower titers, at which expression of transgenes is very specific, there is still minimal non-specific expression that is not detected with immunohistochemistry. This possibility could be examined by using different detection methods, such as *in situ* hybridization.

When decreasing the titer to reach highly specific expression, eYFP expression in MCH neurons decreased, meaning that the regulation of eYFP expression is lower, which could potentially decrease the intensity of neuronal activation when light activates ChR2 and have implications for the magnitude of experimental outcomes (Fig. 3). We also showed that eYFP was expressed at lower levels in MCH^−^ cells compared to MCH^+^ cells in rats injected with MCH-ChR2:eYFP at high titer. Therefore, when MCH^+^ and MCH^−^ cells are activated with optogenetics, manipulation of MCH^−^ cell activity might be negligible. We therefore determined whether cells that are targeted non-specifically express sufficient amounts of cDNA to be manipulated. In rats expressing Gq:mCherry under the control of the MCH promoter in MCH^+^ and MCH^−^ cells, CNO increased cFos immunoreactivity in both MCH^−^ and MCH^+^ cells. Therefore, even though expression of transgenes is lower in MCH^−^ cells, this may result in their activation and contribute to the observed effects of chemogenetic or optogenetic stimulation.

Moreover, in Fig. 4, we aimed to target MCH neurons in the lateral hypothalamus of rats by using a combination of MCH-Cre and Cre-dependent (DIO-)ChR2:eYFP. We observed non-specific expression which was not affected by lowering the titer, whereas the efficiency to target MCH neurons was decreased. Since, theoretically, one molecule of Cre is sufficient to recombine many molecules of DIO-ChR2:eYFP, lowering the titer of Cre did not impact specificity of ChR2, although efficiency to target MCH neurons decreased. For these reasons, we conclude that this strategy is not successful.

We conclude that proper targeting of cDNAs using short cell-specific promoters in viral vectors depends on the type of promoter and the titer of injection. We recommend that promoters such as the one of the orexin gene, should be optimized further to reach target specificity. Established promoters, like the MCH promoter, should be used with caution since at high titers off-target expression occurs, which may impact specificity of the intended manipulation.

## Methods

### Animals

Adult wistar (Crl:WU), Long-Evans rats and adult C57Bl/6J and R26R-EYFP (L-YFP) mice (006148, Jackson laboratories, Bar Harbor, ME, US) on C57Bl/6J background were used. Animals were housed socially and kept under a normal 12:12 hr light-dark cycle with lights off at 19:00. All animals were kept at room temperature (21 ± 2 °C) and 40–60% of humidity conditions. They were fed with chow and water *ad libitum*. All experiments were approved by the Animal Ethics Committee of Utrecht University and conducted in agreement with Dutch laws (Wet op de Dierproeven, 1996; revised 2014) and European regulations (Guideline 86/609/EEC; Directive 2010/63/EU).

### Plasmid construction and Viruses

To create pAAV-MCH-ChR2:eYFP, PCR with forward 5′-TTAGACGCGTTCTAGAGATAACTTCTATTTAATAAGG-3′ and reverse 5′-GTACGGATCCCCTGTTTGCTGCTCCGTAAAGCCGAAG-3′ primers was performed on rat genomic DNA in order to amplify the rat 463 bp-long promoter of the preproMCH gene^8^. The PCR product was ligated into pGEMT.easy (Promega, Madison, WI, US) (pGEMT-MluI-MCH-BamHI) and sequenced with Sanger sequencing. Plasmids pGEMT-MluI-MCH-BamHI and pAAV-CaMKIIa-hChR2(C128S/D156A)-EYFP (gift from Karl Deisseroth, Addgene, Watertown, MA, US, plasmid # 35501) were digested with MluI and BamHI and the insert MCH was ligated into backbone pAAV-hChR2(C128S/D156A)-EYFP.

To create pAAV-MCH-Gq:mCherry, PCR with forward 5′-ACGCGTTCTAGAGATAACTTCTATT-3′ and reverse 5′-GTCGACGGATCCCCTGTTTGCTGCTC-3′ primers was performed on pGEMT-MCH (see above). The PCR product was ligated into pGEMT.easy and sequenced with Sanger sequencing. The latter plasmid together with pAAV-hSyn-hM3D(Gq)-mCherry (a gift from Bryan Roth, Addgene plasmid # 50474) were digested with MluI and SalI and the insert MCH was ligated into backbone pAAV-hM3D(Gq)-mCherry.

To create pAAV-Ore-ChR2:eYFP, the human 1.3 kb orexin promoter^13^ was cut off with MluI and BamHI from plasmid pAAV-hOrexin-tdTomato (gift from Takeshi Sakurai^13^) and ligated into backbone pAAV-hChR2(C128S/D156A)-EYFP.

To create pAAV-MCH-Cre, plasmids pGEMT-MluI-MCH-BamHI - created above - and pAAV-EF1a-Cre (gift from Karl Deisseroth, addgene plasmid # 55636) were digested with MluI and BamHI and the insert MCH was ligated into the backbone pAAV-Cre.

To create pAAV-Oxt-Cre, PCR with forward 5′-ACGCGTCACAGCAGGTTCTAATACAGAGTTT-3′ and reverse 5′-GGATCCGGTACCGGCGATGGTGCTCAGT-3′ primers was performed on mouse genomic DNA in order to amplify the mouse 600 bp-long Oxytocin promoter^11^. The PCR product was ligated into pGEMT.easy and sequenced with Sanger sequencing (pGEMT-Oxt). pGEMT-Oxt and pAAV-EF1a-Cre were digested with MluI and BamHI and the insert Oxt was ligated into the backbone pAAV-Cre.

All created pAAV plasmids were maxi-prepped and the sequence between the inverted terminal repeats (ITRs) was confirmed with Sanger sequencing.

Serotype 5 AAV viruses were generated as described earlier^15^, except that each plasmid was co-transfected with the pDP5 plasmid^20^ at a molar ratio of 1:1, resulting in AAV vectors rAAV5-MCH-ChR2:eYFP (MCH-ChR2:eYFP), rAAV5-MCH-hM3D(Gq)-mCherry (MCH-Gq:mCherry), rAAV5-Ore-ChR2:eYFP (Ore-ChR2:eYFP), rAAV5-MCH-Cre (MCH-Cre) and rAAV5-Oxt-Cre (Oxt-Cre). All viruses were dissolved in sterile PBS 1x with 5% glycerol in low adhesion PCR tubes (K77301, BIOplastics, Landgraaf, the Netherlands) and 10 µL aliquots were frozen at −80. Titer was determined on the high titer stock once using real-time PCR (qPCR) with primers binding on the wPRE element. We did not determine titer of diluted stocks as we used low adhesion tubes, however we cannot exclude that the titer of diluted stocks was lower than calculated due to stickiness of the virus to tubing.

Viral vector rAAV5-EF1a-DIO-hChr2(H134R)-EYFP (DIO-ChR2:eYFP) was purchased from UNC Vector Core (Chapel Hill, NC, US).

### Stereotaxic surgeries

On the day of the surgeries the virus aliquots were thawed on ice and serial dilutions were made with sterile PBS 1x. All aliquots were kept on ice until loaded onto the syringe. Rats were anesthetized with an intramuscular injection of hypnorm (0.315 mg/kg fentanyl and 10 mg/kg fluanisone, Janssen Pharmaceutica, Beerse, Belgium). Mice were anesthetized with ketamine (75 mg/kg, Narketan, Vetoquinol BV, Breda, The Netherlands) and medetomidine (1 mg/kg, Sedastart, AST Farma BV, Oudewater, The Netherlands). Animals were given eye cream (CAF, CEVA Sante Animale BW, Naaldwijk, The Netherlands) and were placed on a stereotaxic apparatus (David Kopf Instruments, Tujunga, USA or Configuration Stereotaxic, 68U017, UNO, The Netherlands). A small incision was made along the midline of the skull and additional analgesia was applied by spraying Xylocaine (lidocaine 100 mg/ml, AstraZeneca BV, The Hague, The Netherlands) on the skull. Per surgery, a maximum of 3 animals per titer were injected (~1 hour), during which the virus was kept at room temperature in the tubing. Viruses were injected unilaterally using a 34 G stainless steel needle connected to 10 ul Hamilton syringe at a rate of 0.05–0.10 μL/min. In 3 batches of C57Bl/6J mice, 0,2 μL of Ore-ChR2:eYFP was injected in the hypothalamus (−1.30  anteroposterior (AP), ±1.80 mm mediolateral (ML) from Bregma, and −5.40 mm dorsoventral (DV) from the skull, at an angle of 10°). In 2 batches of L-YFP mice, 0,2 μL of Oxt-Cre was injected in the paraventricular nucleus (PVN) (−0.80  AP, ±0.70 mm ML from Bregma, and −5.00 mm DV from the skull, at an angle of 5°). In 5 batches of Wistar rats, 0,5 μL of MCH-ChR2:eYFP or MCH-Cre mixed with DIO-ChR2:eYFP were injected in the lateral hypothalamus (−2.90  AP, ±1.80 mm ML from Bregma, and −9.40 mm DV from the skull, no angle). In 2 batches of Long-Evans rats, 0,5 μL of MCH-Gq:mCherry was injected in the lateral hypothalamus (−2.80  AP, ±1.60 mm ML from Bregma, and −9.40 mm DV from the skull, no angle). Groups were balanced between different batches. In a Wistar rat, 1 μL of MCH-ChR2 was injected in the ventral tegmental area (VTA) (−5.60  AP, ±1.30 mm ML from Bregma, and −8.20 mm DV from the skull, at an angle of 5°). Titers of viruses injected are shown in Table 1. After injection, the needle was maintained at its injection position for 15 min. After surgery, the animals were given carprofen for pain relief (5 mg/kg per day for 3 days, subcutaneous (s.c.)) and saline (For mice 0.4 ml/10 gr and for rats 1 ml/100 gr, once, s.c.).

**Table 1:** Overview of all the stereotaxic injections performed in this study.

Viral vector	Injected titer (x 10^9^ g.c. per µL)	Injected Volume (μL)	Species	Region
MCH-ChR2:eYFP	3.0	0,5	rat	LH, VTA
	0.3	0,5	rat	LH
	0.1	0,5	rat	LH
	0.03	0,5	rat	LH
Ore-ChR2:eYFP	0.3	0,2	mouse	LH, PeF
	0.1	0,2	mouse	LH, PeF
MCH-Gq:mCherry	0.3	0,5	rat	LH
	0.1	0,5	rat	LH
MCH-Cre	0.1	0,5	rat	LH
	0.03	0,5	rat	LH
	0.01	0,5	rat	LH
	0.003	0,5	rat	LH
Oxt-Cre	0.8	0,2	mouse	PVN
	0.05	0,2	mouse	PVN

### Histology

Two weeks after surgery animals were sacrificed with sodium pentobarbital overdose (200 mg/mL, Euthanimal, Alfasan BV, The Netherlands). Animals were perfused with ice-cold 1x Phospate Buffered Saline (PBS) pH 7.3, followed by ice-cold 4% paraformaldehyde (PFA) in 1x PBS pH 7.3. Brains were removed and incubated overnight in 4% PFA, then transferred consecutively to 20% for 1 day and 30% sucrose solution (in 1x PBS) for 2 days. Brains were snap-frozen by isopentane immersion and stored at −80 °C. Coronal sections were sliced at 40 µm thickness in a cryostat (Leica, Wetzlar, Germany).

### Immunofluorescence

For cFos detection, free-floating sections from rat hypothalamus were incubated for 30 minutes at 60 degrees in Citrate Buffer (10 mM Sodium Citrate, 0.05% Tween 20, pH 6.0), followed by the general immunohistochemical protocol described next. Sections from the rat and mouse hypothalamus or rat VTA were incubated with blocking solution (5% normal goat serum (NGS), 5% normal donkey serum (NDS), 1% Triton X-100 in 1x PBS) for 1 hour at room temperature (RT), followed by overnight incubation at 4 degrees with primary antibodies (see Table 2) in carrier solution (1,5% NGS, 1,5% NDS, 0,25% Triton X-100 in 1x PBS). Sections were then incubated with the secondary antibodies (see Table 2) in carrier solution for 1 hour at RT and in DAPI (1:1000 in PBS 1x) for 15–30 minutes at RT. Between all steps sections were washed 3 times for 5–10 minutes in PBS 1x. Sections were then mounted on microscope glasses, let to dry and covered with Fluorsave (Calbiochem, San Diego, CA, US).

**Table 2:** Information about all the antibodies used in this study.

Antibody	Type	Concentration	Catalogue no.	Vendor	Experiment
rabbit anti-MCH	Primary	1/1000	M8440	Sigma-Aldrich, St. Louis, MO, US	MCH-ChR2, MCH-Gq, MCH-Cre
chicken anti-GFP	Primary	1/750	ab13970	Abcam, Cambridge, UK	MCH-ChR2, Ore-ChR2, MCH-Cre, Oxt-Cre
mouse anti-NeuN	Primary	1/1000	ab104224	Abcam, Cambridge, UK	MCH-ChR2
rabbit anti-Orexin A	Primary	1/1000	ab6214	Abcam, Cambridge, UK	Ore-ChR2
rabbit anti-Oxytocin	Primary	1/1000	ab911	Milipore, Burlington, MA, US	Oxt-Cre
mouse anti-mCherry	Primary	1/1000	632543	Rockland, Limerick, PA, US	MCH-Gq
guinea pig anti-cFos	Primary	1/500	226 004	Synaptic Systems, Göttingen, Germany	MCH-Gq
donkey anti-rabbit 647	Secondary	1/500	A31573	Life Technologies, Carlsbad, CA, US	MCH-ChR2, MCH-Gq, MCH-Cre
goat anti-chicken 488	Secondary	1/500	ab150169	Abcam, Cambridge, UK	MCH-ChR2, Ore-ChR2, MCH-Cre, Oxt-Cre
goat anti-mouse 405	Secondary	1/500	A31553	Life Technologies, Carlsbad, CA, US	MCH-ChR2
goat anti-rabbit 568	Secondary	1/500	ab175471	Abcam, Cambridge, UK	Ore-ChR2, Oxt-Cre
goat anti-mouse 568	Secondary	1/500	ab175473	Abcam, Cambridge, UK	MCH-Gq
goat anti-guinea pig 488	Secondary	1/500	A-11073	Life Technologies, Carlsbad, CA, US	MCH-Gq

### Imaging and image analysis

For quantifications, pictures were taken at 20x magnification at a confocal microscope (Olympus Fluoview FV1000, Olympus, Tokyo, Japan). Regarding MCH and GFP co-localization analysis in the rat lateral hypothalamus, four sections with an interval of 0,2–0,3 mm ranging from −2,6 to −3,8 mm caudal to bregma (as indicated in^21^), were selected from each animal. Regarding Orexin and GFP co-localization analysis in the mouse hypothalamus, four sections with an interval of 0,2–0,3 mm ranging from −1,1 to −2,2 mm caudal to bregma (as indicated in^22^), were selected from each animal. Cells were manually counted using the Cell Counter plugin in ImageJ after blinding.

Specificity was defined as the percentage of eYFP^+^ cells that also stained for MCH or the percentage of eYFP^+^ cells that also stained for orexin or the percentage of mCherry^+^ cells that stained for MCH. Efficiency was defined as the percentage of MCH^+^ cells that stained for eYFP in the LH or the percentage of Orexin^+^ cells that stained for eYFP in the hypothalamus or the percentage of MCH^+^ cells that stained for mCherry in the LH.

Regarding quantifications of intensity, regions of interest (ROIs) with the freehand selection tool of ImageJ were created containing most of the soma of a cell, and mean intensity within the ROIs was automatically measured.

### Drugs

Clozapine-N-oxide (CNO; kindly provided by Bryan Roth and NIMH, Bethesda, MD, US or purchased from AK scientific, Cat. No. AMTA056, Union City, CA, US) was dissolved in sterile saline (0.9% NaCl). All injections were given intra-peritoneally (i.p.) in rats at 1 ml per kg body weight.

### Injections of CNO in MCH-Gq:mCherry rats

Rats were administered with CNO i.p. at 1 mg/kg or Saline (0,9% NaCl) at 07:00 (start of the light phase). Food and water was removed from the cages and 2 hours later rats were sacrificed as described above.

### Statistical analyses

Statistical analyses were performed with GraphPad Prism 7.0 (Graphpad Software, San Diego, CA, US). Data was checked for normality and nonparametric tests were performed when data did not follow a Gaussian distribution. Graphs of nonparametric data are presented as median instead of mean, since mean is not the proper measure of the central tendency of data with nonparametric distribution.

## Supplementary information

Supplementary figures
