# Supplementary material for: Considerations related to the use of short neuropeptide promoters in viral vectors targeting hypothalamic neurons
Source: Sci Rep. 2019 Jul 31;9:11146. doi: 10.1038/s41598-019-47417-9 (PMC6668470; doi:10.1038/s41598-019-47417-9)
Supplement: Supplementary file 1 — Supplementary figures [file 41598_2019_47417_MOESM1_ESM.pdf]

Considerations related to the use of short neuropeptide promoters in viral vectors targeting hypothalamic neurons

N. Kakava-Georgiadou, C. Bullich-Vilarrubias, M.M. Zwartkruis, M.C.M Luijendijk, K.M. Garner, R.A.H. Adan

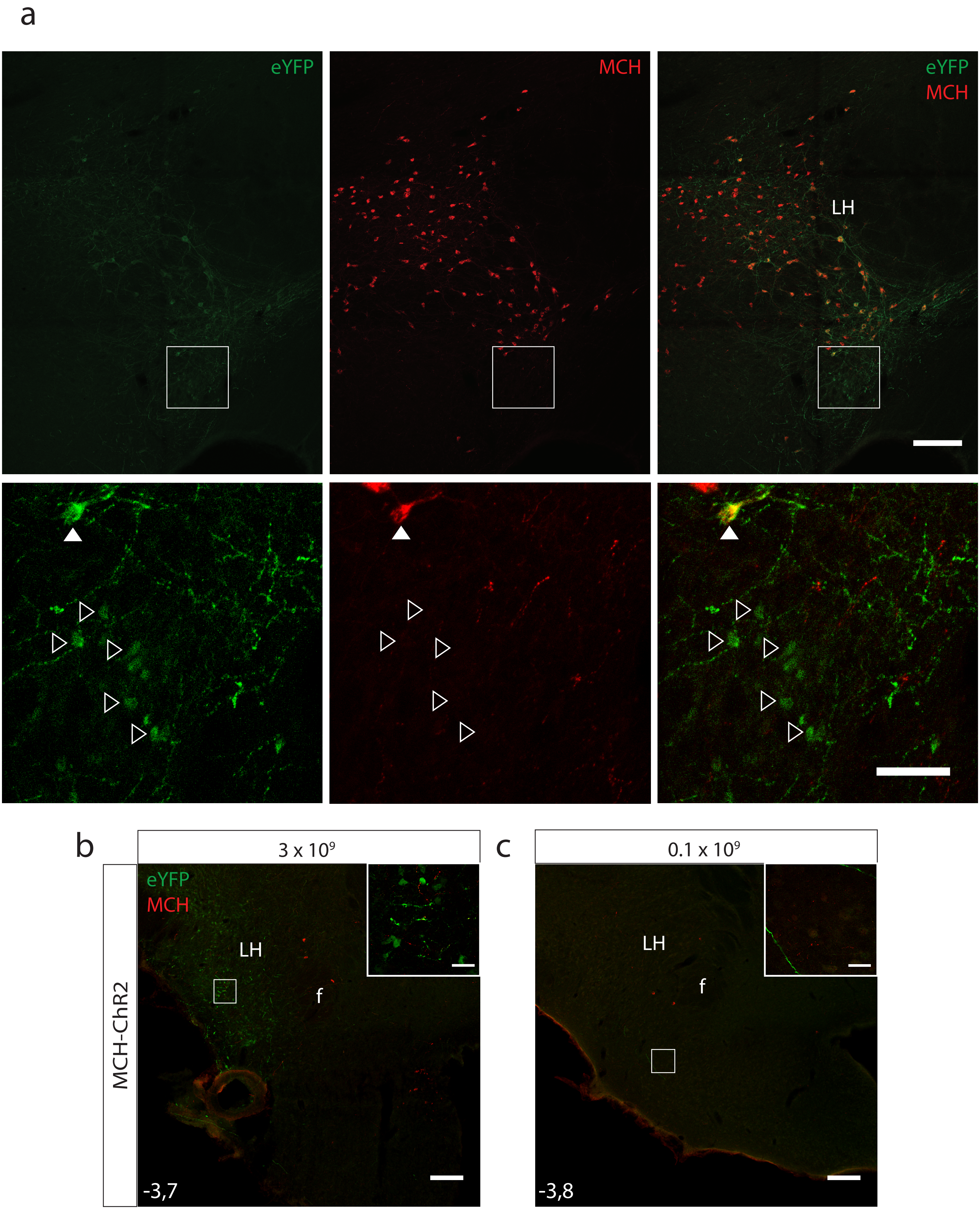

Figure S1

A Rat injected with MCH-ChR2:eYFP in the lateral hypothalamus at 3.0 x 10^9 g.c. per µL; co-staining of ChR2: eYFP (green) and MCH (red); LH=lateral hypothalamus; Scale bars: low magnification pictures: 1 00 µm, high magnification pictures: 30 µm. **B** Rat injected with MCH-ChR2:eYFP in the lateral hypothalamus at 3.0 x 10^9 g.c. per µL; co-staining of ChR2: eYFP (green) and MCH (red), -3,7 mm caudal to bregma; **C** Rat injected with MCH-ChR2:eYFP in the lateral hypothalamus at 0.1 x 10^9 g.c. per µL; co-staining of ChR2:eYFP (green) and MCH (red), -3,8 mm caudal to bregma; f=fornix, LH=lateral hypothalamus; Scale bars: low magnification pictures: 200 µm, high magnification pictures: 30 µm.

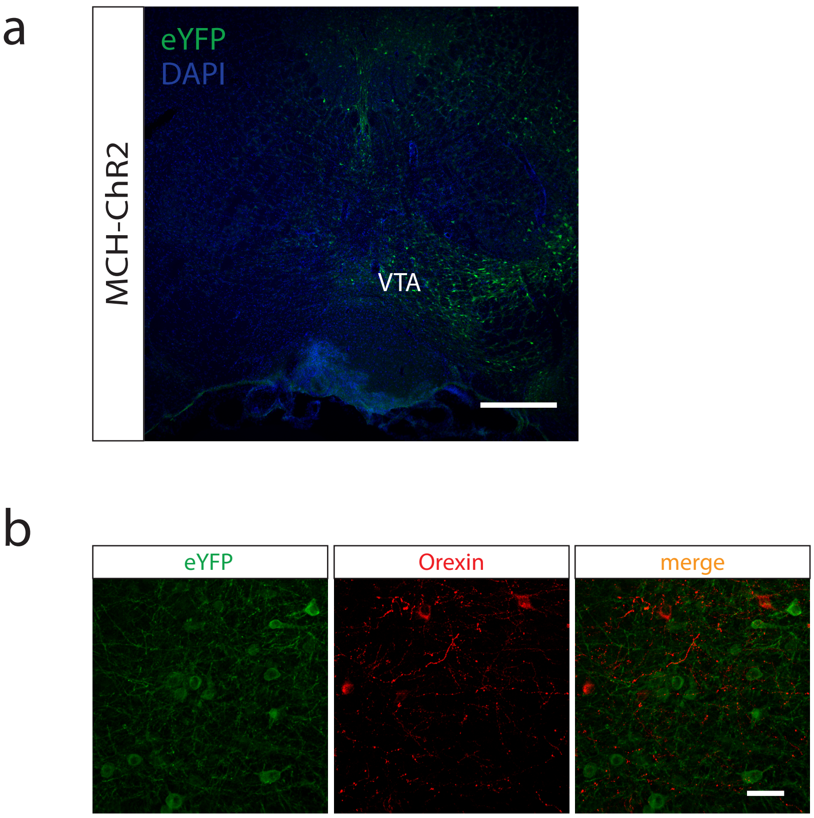

Figure S2
A Rat injected with MCH-ChR2:eYFP in the VTA at 3.0 x 10^9 g.c. per μL; co-staining of ChR2:eYFP (green) and DAPI (blue); **B** Rat injected with MCH-ChR2:eYFP in the lateral hypothalamus at 3.0 x 10^9 g.c. per μL; co-staining of ChR2: eYFP (green) and Orexin (red); Scale bars: A) 500 μm, B) 50 μm

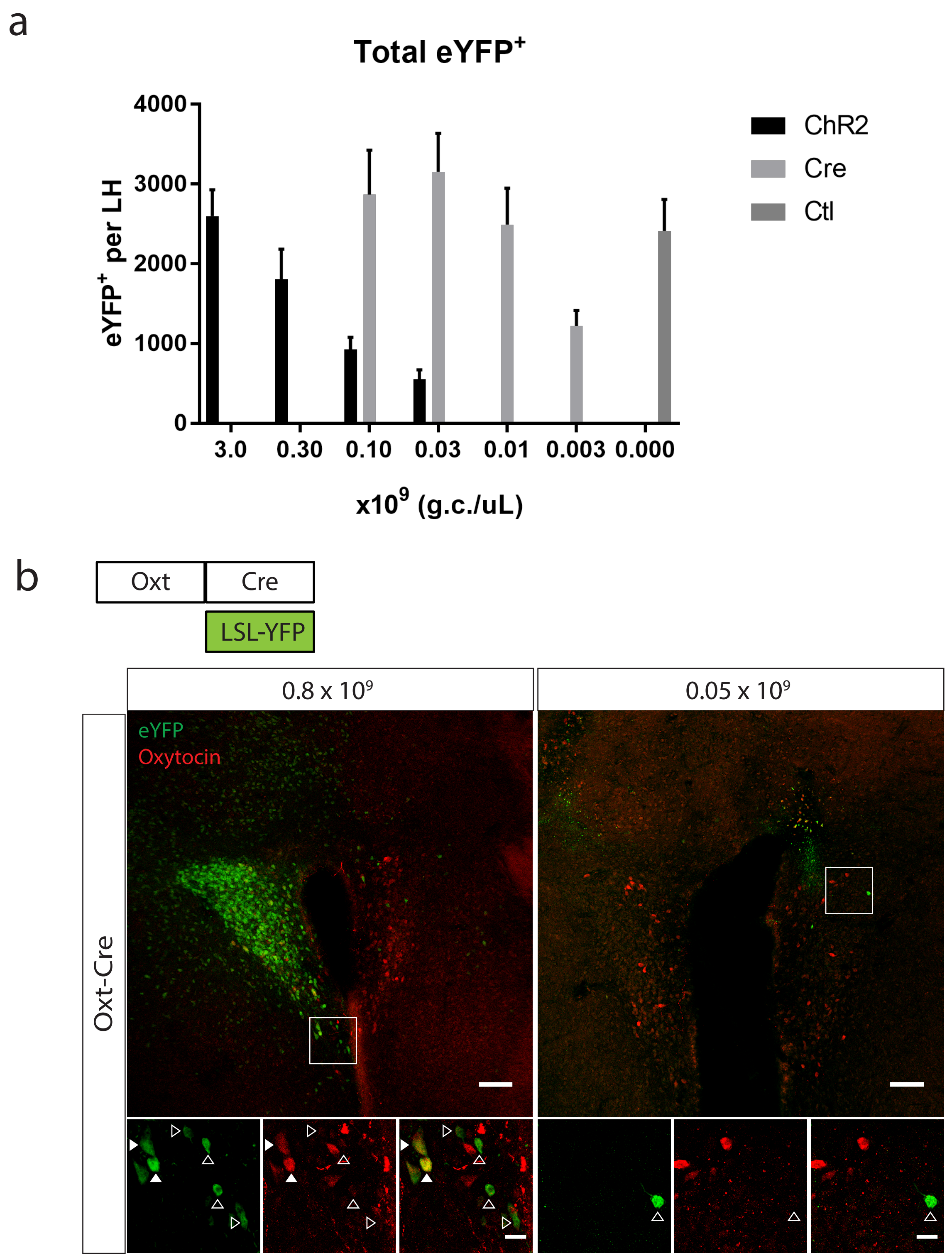

Figure S3
A Total eYFP+ cell counts in the lateral hypothalamus of rats after injections with constructs MCH-ChR2:eYFP (ChR2), MCH-Cre& DIO-ChR2:eYFP (Cre) at various titers and DIO-ChR2 (Ctl) (1.0 x 10^9 g.c. per µL); **B** Oxt-Cre injected in the PVN of Rosa26-LSL-YFP mice; Left: titer of 0.8 x 10^9 g.c. per µL, Right: titer of 0.05 x 10^9 g.c. per µL; co-staining for YFP (green) and Oxytocin (red); YFP expression in Oxytocin$^+$ cells (white full arrows), YFP expression in Oxytocin- cells (white empty arrows); Scale bars: low magnification pictures: 100 µm, high magnification pictures: 10 µm; Error bars represent mean±SEM
